# Noise Eliminated Ensemble Empirical Mode Decomposition Scalogram Analysis for Rotating Machinery Fault Diagnosis

**DOI:** 10.3390/s21238114

**Published:** 2021-12-04

**Authors:** Atik Faysal, Wai Keng Ngui, Meng Hee Lim, Mohd Salman Leong

**Affiliations:** 1 College of Engineering, Universiti Malaysia Pahang, Pekan Pahang 26600, Malaysia; faisal.atik@gmail.com; 2 Institute of Noise and Vibration, Universiti Teknologi Malaysia, Johor Bahru 81310, Malaysia; limmenghee@gmail.com (M.H.L.); salman.leong@gmail.com (M.S.L.)

**Keywords:** convolution neural network, empirical mode decomposition, deep convolution generative adversarial network, continuous wavelet transform

## Abstract

Rotating machinery is one of the major components of industries that suffer from various faults due to the constant workload. Therefore, a fast and reliable fault diagnosis method is essential for machine condition monitoring. In this study, noise eliminated ensemble empirical mode decomposition (NEEEMD) was used for fault feature extraction. A convolution neural network (CNN) classifier was applied for classification because of its feature learning ability. A generalized CNN architecture was proposed to reduce the model training time. A sample size of 64×64×3 pixels RGB scalograms are used as the classifier input. However, CNN requires a large number of training data to achieve high accuracy and robustness. Deep convolution generative adversarial network (DCGAN) was applied for data augmentation during the training phase. To evaluate the effectiveness of the proposed feature extraction method, scalograms from related feature extraction methods such as ensemble empirical mode decomposition (EEMD), complementary EEMD (CEEMD), and continuous wavelet transform (CWT) are classified. The effectiveness of scalograms is also validated by comparing the classifier performance using grayscale samples from the raw vibration signals. All the outputs from bearing and blade fault classifiers showed that scalogram samples from the proposed NEEEMD method obtained the highest accuracy, sensitivity, and robustness using CNN. DCGAN was applied with the proposed NEEEMD scalograms to further increase the CNN classifier’s performance and identify the optimal number of training data. After training the classifier using augmented samples, the results showed that the classifier obtained even higher validation and test accuracy with greater robustness. The proposed method can be used as a more generalized and robust method for rotating machinery fault diagnosis.

## 1. Introduction

When any fault occurs in a rotating machinery part, the vibration data carry the fault information in a series of periodic impulses. Due to the presence of various signals and noise in the environment, the fault information in raw vibration data may be easily submerged in the noise. Thus, the separation of faulty information might get complicated. Generally, signal-processing methods are applied to extract the tiniest information possible. Typically, the baseline condition has smaller impulses, and fault conditions have relatively higher impulses, making them easier to differentiate. However, in the practical field, the search for the fault occurrence in the whole signal is unfeasible by human convention. Thus, the use of artificial intelligence for finding the fault pattern in the signal is a popular method [1]. Artificial intelligence or machine learning methods offer automated fault diagnosis by learning from the fault features from the previous data.

Typically, signal processing requires skilled human interaction to select tunable parameters during preprocessing. The time-frequency adaptive decomposition methods can automatically decompose a signal without skilled human intervention. A robust time-frequency analysis, empirical mode decomposition (EMD), was first presented in 1998. Since its arrival, EMD has been applied to stationery and non-stationary signals in numerous fault diagnosis research [2,3]. In EMD, the time scale is divided into a set of orthogonal components known as intrinsic mode function (IMF). In papers [4,5], the authors used EMD and EEMD for bearings and gears fault diagnosis. Nguyen et al. [6] proposed a novel fault diagnosis technique for rolling bearing. A combination of EMD, NB classifier, and noise components threshold was suggested for their approach. However, the major drawback is the mode mixing of the IMFs.

To solve the mode-mixing problem, Huang and Wu [7] proposed the EEMD, which can overcome the limitations of EMD. Wu et al. [8] added an autoregressive model with EEMD to detect looseness faults of rotor systems. EEMD-based methods were proposed by Lei et al. [9] to identify initial rub-impact faults of rotors and compared EEMD with EMD to show superiority. Nonetheless, white noise can still be highly present in the IMFs of EEMD after even performing a bunch of ensembles. To significantly decrease the white noise, many ensembles are required that enhance the computational cost. Yeh et al. [10] proposed CEEMD to reduce the limitations of EEMD. CEEMD adds white noise in positive and negative pairs that can reduce the residual noise in the reconstructed signal with fewer ensembles. Yang [11] combined CEEMD and wavelet threshold for noise reduction of rolling bearing vibration signals. IMFs were obtained using CEEMD and selected based on kurtosis index and correlation coefficients. The fault impulses were obtained using the envelope spectrum. CEEMD can significantly decrease white noise, but it does not eliminate the limitations and needs further attention. To address this problem, Faysal et al. [12] proposed noise eliminated EEMD, which reduces the white noise in the IMFs while maintaining the same number of ensembles. In this method, the ensemble white noise is also decomposed and subtracted from the original ensemble IMFs. Therefore, more white noise can be eliminated from the resulting IMFs.

Lately, deep learning has become the most attractive research trend in the area of AI. With the ability to learn features from raw data by deep architectures with many layers of non-linear data processing units, the deep neural network (DNN) model is a promising tool for intelligent fault diagnosis. In supervised learning algorithms, the convolution layer model is the most influential architecture for computer vision and pattern recognition [13]. Generally, the CNN architecture is a handy tool for image processing and learning features from the input images. The wavelet representation of images can be an ideal input because it can extract the most information from 1D time-domain signals and represent them with both time and frequency information. The wavelet images can be presented as spectrogram or scalogram plots [14]. Kumar et al. applied grayscale spectrogram images from analytical WT as the input of CNN for centrifugal pump defects identification [15]. Analysis showed that the proposed improved CNN significantly improved identification accuracy by about 3.2% over traditional CNN. Nevertheless, compared to the spectrogram representations, which produce a constant resolution, a scalogram approach is more suitable for the chore of fault classification due to its detailed depiction of the signal. Wavelet scalogram representations have been proven effective and gaining more popularity over spectrogram representation [16]. Scalogram is defined as the time-frequency representation that depicts the obtained energy density using CWT [17]. Verstraete et al. applied CNN on two public bearing datasets for fault diagnosis [18]. Three different types of input images were produced, namely, STFT spectrogram, wavelet scalogram, and HHT image. Comparing the output accuracy from all the image types, the wavelet scalogram appeared to be the best fit. The previous research shows that the wavelet scalograms have a much higher advantage than the other image representations.

As the years went by, the CNN architecture has also increased in its size and layers to obtain better performance [19,20,21]. Although these models perform with good accuracy, their training time is very high, which is a high price for a small improvement. Moreover, CNN can be computationally cumbersome and not all the processing units can afford that. The embedded system can only process up to a certain limit; on the other hand, a quick processing system is anticipated in the practical fields. For this reason, researchers have been more focused on a lightweight CNN model without much compromise of accuracy. A lightweight CNN consists of only a few convolution layers and fully connected layers. Therefore, it uses less random-access memory and processing units. Fang et al. used two CNN models, where the first one was a 1D CNN to extract the multichannel features from the original signal [22]. Later, a lightweight CNN was applied where the weights were adjusted via a spatial attention mechanism to classify the features. The experimental results demonstrate that the proposed method has excellent anti-noise ability and domain adaptability. Thus, it is a widespread practice in fault diagnosis research to choose a lightweight CNN model over a very deep CNN for faster model training.

Although CNN is very handy in image processing, a downside is that this classifier requires massive training data to generalize well. Several data augmentation techniques are available to introduce more data during the training phase. The most common ones are (1) geometrical transformation and (2) the addition of white noise. In geometrical transformation, the image is rotated, flipped, adjusted brightness, transitioned, and cropped to augment new samples [23]. However, this approach would not work well with the wavelet scalograms because scalograms are a graphical representation, unlike images of objects. A transformed representation of a graph would have a new meaning and may result in reduced accuracy. In the second approach of white noise addition, some degree of noise is added with the original signal to introduce some abnormality in the training data. This technique works well because the test data might be noisy in the practical field and differ considerably from the training data. However, white noise addition is not the best approach for the ensemble algorithms, such as EEMD, CEEMD, and NEEEMD, because these algorithms already use ensemble noise to reduce the white noise.

One of the most impressive and arising techniques in deep learning, generative adversarial networks (GANs), has been getting much attention ever since its arrival [24]. GANs is an unsupervised learning algorithm used for image generation where it takes some real images and produces similar outputs from complete white noise. Thus, GANs can be used for data augmentation, where the augmented data never existed before, and it saves the classifier from repeated training samples. Arjovsky proposed Wasserstein GAN, which is a big step towards the improvement of GANs to produce realistic images [25]. However, WGAN works best for medical images, and the performance of images generated from signals is unknown. Wang proposed a fault diagnosis method for planetary gearbox by combining GANs and autoencoder [26]. However, as it uses the vanilla GANs, the output is comparatively noisy, and the use of autoencoders can lose necessary data, which needs additional research. Radford proposed DCGAN, which uses convolution instead of dense layers and produces high-resolution hyper-realistic images [27]. Since its arrival, DCGAN has been a popular tool for data augmentation in medical imaging and fault diagnosis fields. A method called FaultFace was proposed for ball-bearing joint failure detection, which uses a 2D representation of time-frequency signals from an unbalanced dataset [28]. DCGAN was applied to generate more 2D samples for employing a balanced dataset. The obtained output proves that the FaultFace strategy can achieve good performance for an unbalanced dataset. Liang used wavelet image samples of different bearing and gearbox load conditions as the input of CNN classifier [29]. After using the augmented image samples from DCGAN, the classifier performed more accurately for various load conditions and obtained higher robustness. Therefore, DCGAN appears to be an ideal fit for GAN-based data augmentation using vibration signals.

## 2. Proposed Method

**Figure 1:**
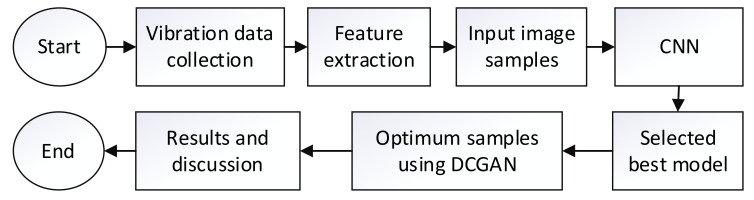
The complete research flow chart.

The proposed fault diagnosis method consists primarily of feature extraction, classification, and data augmentation for optimum training samples selection. The feature extraction process includes input generation from grayscale images and scalogram images. The grayscale samples are generated from the raw signal. The scalogram samples are generated from EEMD, CEEMD, and NEEEMD. Since those image samples are wavelet scalograms, CWT scalograms are also considered for performance comparison. The image samples are classified using CNN. The best-performing model is selected from here based on accuracy and precision. Moreover, the model’s robustness for noisy test samples is also considered to validate the best model selection. Then, DCGAN is applied to produce augmented samples within a range for the best-performing model. The optimum training samples are identified via this process. The model trained on optimum training samples is compared with the previous one to compare the performance improvement. The flowchart of the proposed method is illustrated in Figure 1.

The key contributions of this study are:

1. This work utilizes an improved algorithm named NEEEMD to incorporate NEEEMD features with supervised classification for automated fault diagnosis. This study proves the effectiveness of NEEEMD as a feature extraction algorithm.
2. The IMFs were concatenated to generate a single scalogram image containing all the features from all the IMFs. Therefore, the need for multiple input channels in a CNN classifier was avoided, reducing significant computational complexity.
3. A generalized CNN model that contains four convolution layers was proposed for all the bearing and blade classification models. The proposed classifier contains considerably fewer parameters than many state-of-the-art deep neural network models. However, it can still obtain very impressive accuracy for both bearing and blade datasets.
4. The power of DCGAN was utilized to generate augmented samples in order to supply more training data. DCGAN helped to generate augmented samples on demand and assisted in finding the optimum training samples.

## 3. Data Collection

Two different datasets were used in this study to validate the proposed method. The first dataset is from the Case Western Reserve University bearing dataset [30]. The second dataset is turbine blade data from Universiti Teknology Malaysia [31]. They are described chronologically in the following sub-sections. All the programming materials applied to analyze the datasets of this study are open for public usage (refer to Supplementary Materials).

### 3.1. Bearing Dataset

**Figure 2:**
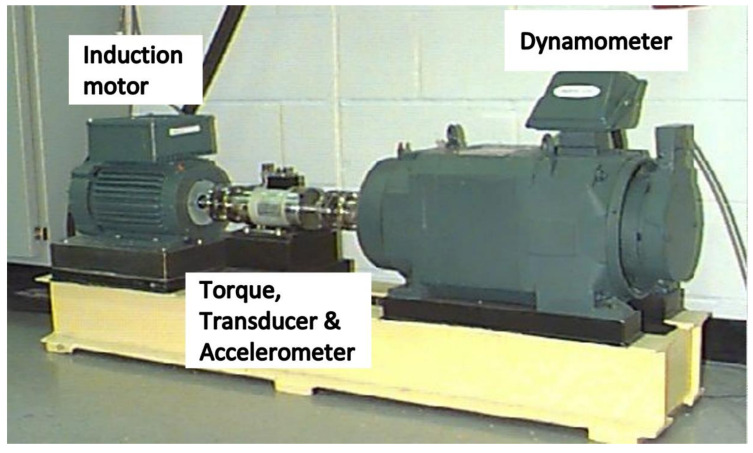
Experimental setup for the bearing data collection.

**Figure 3:**
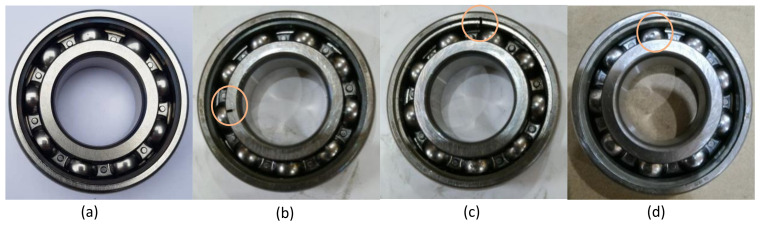
Four bearing conditions (**a**) normal condition, (**b**) inner race fault, (**c**) outer race fault, (**d**) ball fault.

In the bearing dataset, the acceleration data was measured from a 2-hp reliance electric motor bearings placed at the 12 o’clock position. Drive end data were taken from bearing model SKF 6205-2RS JEM. The sampling frequency was 12 kHz. The rotor shaft’s rotating speed was considered 1797 rpm, and the motor load was 0 hp. The setup of the test rig is shown in Figure 2. The bearing dataset mainly has three different fault types at various severity. These faults are inner race fault, outer race fault, and ball fault. Another fault-free condition is the normal condition. A visual representation for these four conditions is presented in Figure 3.

### 3.2. Blade Dataset

**Figure 4:**
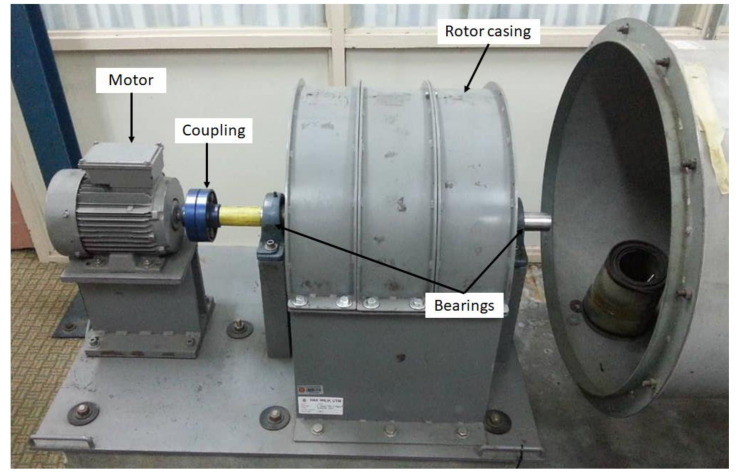
Test rig to simulate blade fault.

The dataset contains vibration signals obtained from the rotor blades operating under constant rotational speed conditions. All blade fault conditions were measured with the sampling rate was 5 kHz for all states, and the rotating speed was set to 1200 rpm (20 Hz). The blade fault simulated in a test rig to collect vibration signals is shown in Figure 4. A three-phase synchronous motor was used in the test rig to control the rotational speed during the experiment.

**Figure 5:**
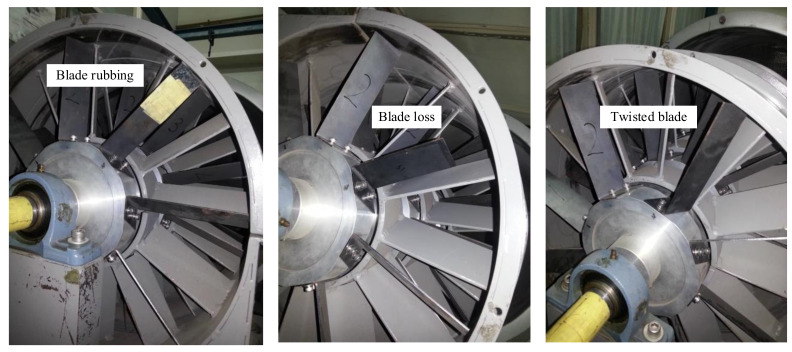
Different blade fault conditions.

Blade rubbing (BR) fault was artificially added in the test rig by adding a metal sheet to a regular blade and thus increasing its length. A 1 mm thick metal sheet was used as the rubbing medium. Because of its hardy characteristics, it prevents wearing out of the rotor casing. The blade rubbing condition was introduced such that the blade edge slightly touched the rotor casing’s inner surface. The blade loss (BL) fault was included by replacing a normal blade with a blade of partial loss. For this experiment, a blade with one-fourth loss was used as a rotor blade in the first, second, and third rows. A normal blade was replaced with another tightened blade in the opposite direction set into the rotor disk to introduce the blade twist (BT) fault. For this work, an artificially induced single twisted blade fault condition was added to the different rows of the rotor blade in the test rig. All the blade fault conditions are presented in Figure 5.

## 4. Classification Models

In this study, mainly two different classification models are developed using bearing and the blade dataset. The bearing dataset has only one classification model, and the blade dataset contains three classification models. A detailed description of those models is provided in the following subsection.

### 4.1. Bearing Fault Classification

Collected vibration signals include the following operating conditions: (1) normal condition, (2) inner race fault, (3) ball fault, and (4) outer race fault. Each fault condition includes three different fault sizes, 0.007, 0.014, and 0.021 inches in diameter. In total, 10 different conditions (1 normal, 9 fault conditions) were considered for the fault diagnosis, and the fault categories are presented in Table 1.

In this work, 600 data points are taken in each bearing sample. The bearing dataset has 10 different classes and 200 samples in each class. For each class, the data are partitioned into train, validation and test set in such a way that the train, validation and test set have 50%, 25%, 25% data, respectively. The training set has 100 samples to train the classifier model, and the validation set has 50 samples to observe and maintain the performance of the training set. Later, 50 testing samples are used to measure the performance of the classifier. For 10 classes in total, the classifier consists of 2000 samples, where 1000, 500, and 500 are for train, validation, and test, respectively.

### 4.2. Blade Fault Classification

In the blade fault test rig, three different blade faults were induced in different rotor rows. A total of 21 different blade fault conditions based on the fault type and rotor location were examined in this study. The blade data are divided into two different categories for classification: (1) Fault diagnosis: 3-class classification, (2) fault localization: 7-class classification. A detailed description of each category is provided in Table 2 and Table 3. Here, R(number) represents the row location of fault occurrence for the particular dataset.

The 3-class fault diagnosis classification has 7 sets of fault data in each class and 21 total sets of data for the whole classifier. The total train, validation, and test data for this model are 5250, 1575, and 1575. Each class contains 1750, 525, and 525 train, validation and test samples.

The 7-class fault localize classification has 3 sets of fault data in each class and 21 total datasets for the whole classifier. The total train, validation, and test data for this model are 5250, 1575, and 1575. Therefore, each class contains 750, 225, and 225 train, validation, and test samples.

## 5. NEEEMD

The NEEMD takes a different approach than CEEMD to eliminate the white noise in the final stage. Instead of adding a negative white noise at the primary stage, it subtracts the IMFs of the same white noise from the final IMFs. The steps of NEEEMD are followings:

1. Add ensemble white noise $w_{i}\left(t\right)$ (whose length is the same as the original signal with a mean of 0 and the standard deviation of 1 to the original signal $X\left(t\right)$ and obtain $X_{i}\left(t\right)$.
2. Decompose $X_{i}\left(t\right)$ using EMD (see Appendix A) [32] and obtain the ensemble means of the IMFs, $c_{j}\left(t\right)$.
  (1) $$c_{j}\left(t\right)=\frac{1}{M}\overset{M}{\underset{i=1}{\sum }}c_{ij}\left(t\right)$$
3. Take the input ensemble white noise $w_{i}\left(t\right)$ and apply EMD on each one of it.
  (2) $$W_{i}=\overset{N}{\underset{j=1}{\sum }}wc_{ij}\left(t\right)+wr_{i}\left(t\right)$$
  where j=1,2,…,N, N is the number of IMFs and $wc_{ij}\left(t\right)$ is the IMFs of noise ($c_{i1},\;c_{i2},\ldots ,c_{iN}$). $wr_{i}\left(t\right)$ denotes the residue of the ith trail.
4. Compute the ensemble means of the IMFs for the noise.
  (3) $$wc_{j}\left(t\right)=\frac{1}{M}\overset{M}{\underset{i=1}{\sum }}wc_{ij}\left(t\right)$$

Subtract the IMFs of noise from the IMFs obtained from EEMD for the reduction of white noise.

(4) $$IMF_{j}=c_{j}\left(t\right)-wc_{j}\left(t\right)$$

5. The original signal can be obtained such that,
  (5) $$X\left(t\right)=\overset{M}{\underset{i=1}{\sum }}IMF_{ij}\left(t\right)+r_{Mj}\left(t\right)-wr_{Mj}\left(t\right)$$
  where $wr_{M}\left(t\right)$ is the residue of the white noise.

**Figure 6:**
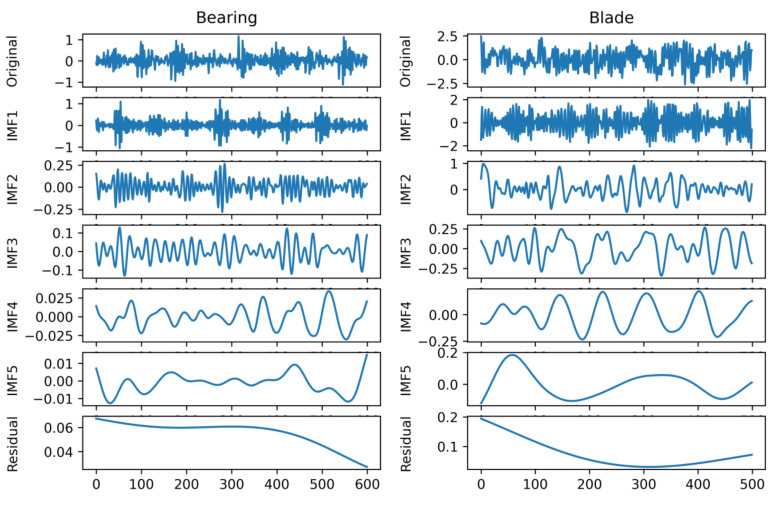
Decomposition results from NEEEMD.

Decomposing the signal provides several IMFs and a residual signal, which is a monotonous signal. For visualization, one random sample from the bearing and blade signal is decomposed using NEEEMD. Figure 6 represents the outputs of NEEEMD for both bearing and blade samples. Here, no mode mixing phenomena were observed in the IMFs. Moreover, the residuals were monotonous, containing virtually no necessary information.

**Figure 7:**
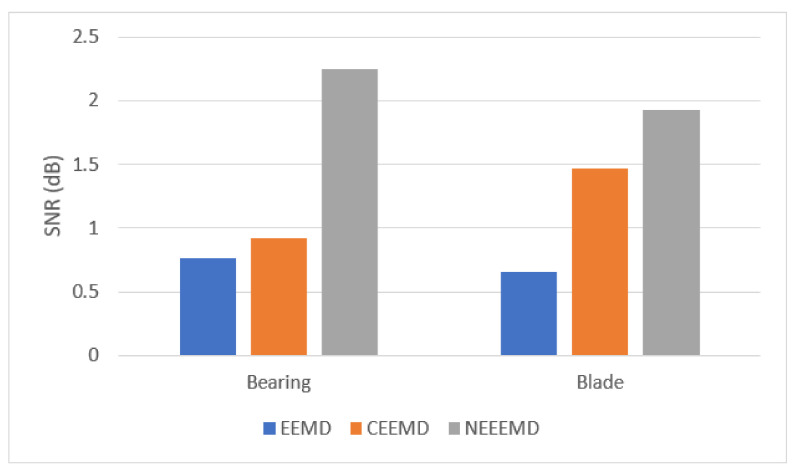
SNR values for reconstructed signals of bearing and blade samples.

It is necessary to prove the superiority of NEEEMD over EEMD and CEEMD statistically. All the statistical analyses of the bearing and blade datasets show a similar trend for EEMD, CEEMD, and NEEEMD. However, only one random sample from the bearing and blade datasets is presented in this study due to space constraints. The final IMFs of NEEEMD are supposed to have less white noise presence than EEMD and CEEMD. To illustrate the effect of reduced white noise, signal-to-noise ratio (SNR) is considered. SNR is computed using the original and reconstructed signals, where the reconstructed signal is the sum of the resulting IMFs. The formula for computing SNR is provided in Equation (6). It is the ratio of the power of the original signal to the power of the reconstructed signal. The SNR of NEEEMD from one random bearing and blade fault sample is compared with EEMD and CEEMD. The outputs are presented in Figure 7. It is seen that in both bearing and blade samples, EEMD obtained the lowest SNR value. CEEMD obtained higher SNR than EEMD, whereas NEEEMD obtained the highest SNR. Therefore, the reconstructed signal from NEEEMD has less noise in it than EEMD and CEEMD.

(6) $$SNR_{dB}=10log_{10}\left(\frac{P_{original\;signal}}{P_{reconstructed\;signal}}\right)=10log_{10}\left(\frac{P_{\left(X\left(t\right)\right)}}{P_{\sum_{i=1}^{M}IMF_{ij}\left(t\right)+r_{Mj}\left(t\right)-wr_{Mj}\left(t\right)}}\right)$$

A frequently used statistical evaluation parameter, RRMSE, is implemented to calculate the restoration error to further emphasize the proposed method’s effect. The ratio between the root-mean-square of the original and reference signal to the root-mean-square of the reference signal is defined as RRMSE [33]. The equation for RRMSE is presented as follows:

(7) $$RRMSE=\frac{||a-b||}{b}$$

Here, a is the original signal, and b is the reconstructed signal.

**Figure 8:**
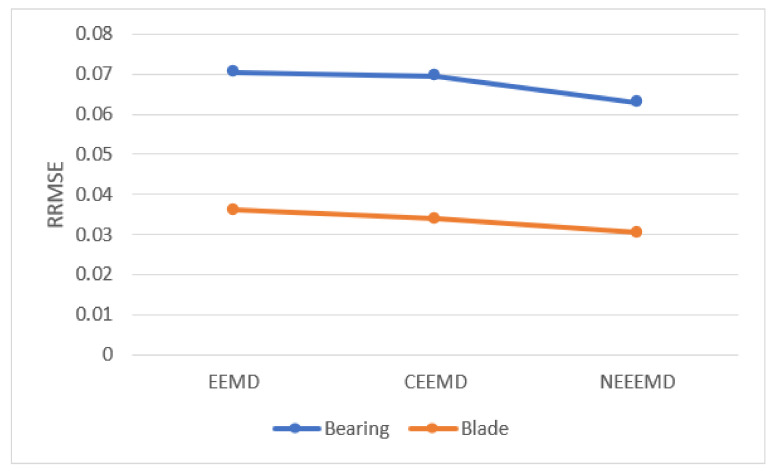
RRMSE values of the bearing and blade samples.

Figure 8 represents the RRMSE values from a random bearing and sample. EEMD obtained higher RRMSE values, 0.0705 for bearing, and 0.0361 for blade sample. CEEMD had a lower reconstructed error than EEMD, 0.0695 and 0.0339 for bearing and blade, respectively. NEEEMD achieved the lowest RRMSE, which are 0.0629 and 0.0304 for bearing and blade. Therefore, NEEEMD has a much lower reconstruction error than EEMD and CEEMD, and proves to be more effective than those previous two improvements.

Next, the signal strength is considered to evaluate the degree of information the reconstructed signal carries. The kurtosis value is an excellent parameter for measuring the signal strength. Wang et al. [34] used the multiplication of the kurtosis in the time and the envelope spectrum domain, which can be applied to determine the strength of the signal. The method is called TESK and is defined as:

(8) $$tk=k_{c}.k_{es}$$

where,

(9) $$k_{c}=\frac{E{\left(X\left(t\right)-\mu_{c}\right)}^{4}}{\sigma_{c}^{4}}$$

(10) $$k_{es}=\frac{E{\left(es\left(f\right)-\mu_{es}\right)}^{4}}{\sigma_{es}^{4}}$$

where, $X\left(t\right)$ as the IMF to be analyzed,$\;\sigma_{c}$ as the standard deviation of $X\left(t\right)$, $\mu_{c}$ as the mean of $X\left(t\right)$, $es\left(f\right)$ as the envelope power spectrum of $X\left(t\right)$, $\mu_{es}$ as the mean of $es\left(f\right)$, $\mu_{es}$ as the standard deviation of $es\left(f\right)$ and $E\left(.\right)$ as the expectation operator.

**Figure 9:**
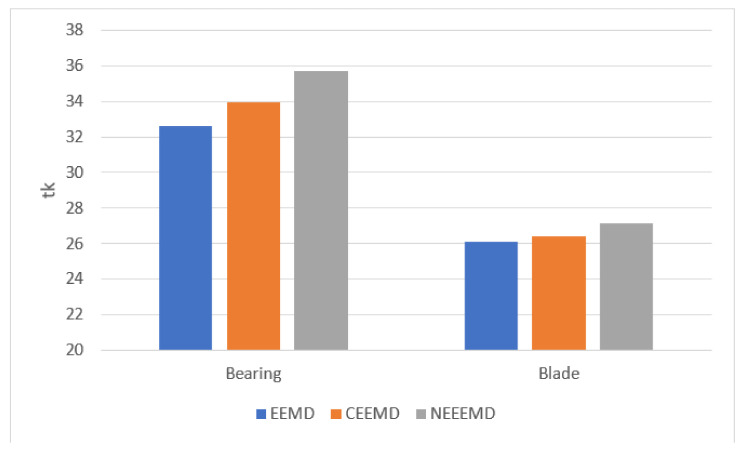
tk values from the bearing and blade samples.

The higher the tk value, the more information the signal contains. Thus, the total value of tk from different signals can be compared to determine the performance of the algorithms. Figure 9 shows the tk values from both bearing and blade samples. For the bearing sample, EEMD has the lowest signal strength, as the tk value is 32.64. The second highest is CEEMD with tk value of 33.97. NEEEMD has the highest signal strength as it obtained the highest tk value, 35.71. The tk values of the blade sample were relatively closer since the sample length is smaller than the bearing sample. Nevertheless, NEEEMD obtained the highest tk value as well. The tk values are 26.08, 26.38, and 27.14 for EEMD, CEEMD, and NEEEMD, respectively. Therefore, in both bearing and blade samples, the NEEEMD signal contains the highest signal strength.

## 6. Features for Deep Learning

The deep learning classifiers in this study work with two types of input samples, namely, grayscale images and scalogram images. The 2D grayscale vibration images are produced from the 1D raw vibration data [35]. The produced image dimension is 64×64 pixels. However, each bearing data sample has 600 data points. Therefore, the samples are converted from a 1×600  vector to a 20×30 matrix and produced the 2D grayscale images. Still, the generated images have a size of 20×30 pixels, so the images are upsampled to produce images of size 64×64. The blade sample length is 500. Therefore, images are upsampled from a 20×25 matrix in this case. Since the images are 2D grayscale, the number of the input channel for CNN, in this case, is 1.

Scalograms are one type of time-frequency representation that uses CWT analysis to obtain the energy density of the signal. A sliding window is used in wavelet representation, which has different resolutions in different regions. CWT decomposes the raw signal into a time scale, which is represented by scaling and translating operations. Morlet wavelet is applied with the time-bandwidth product and symmetry parameter set to 60 and 3, respectively. According to the range of energy of the wavelet in frequency and time, the minimum and maximum scales are automatically determined using 10 voices per octave [36]. Points out of the cone of influence have been handled by the approximation used in MathWorks MATLAB [37].

**Figure 10:**
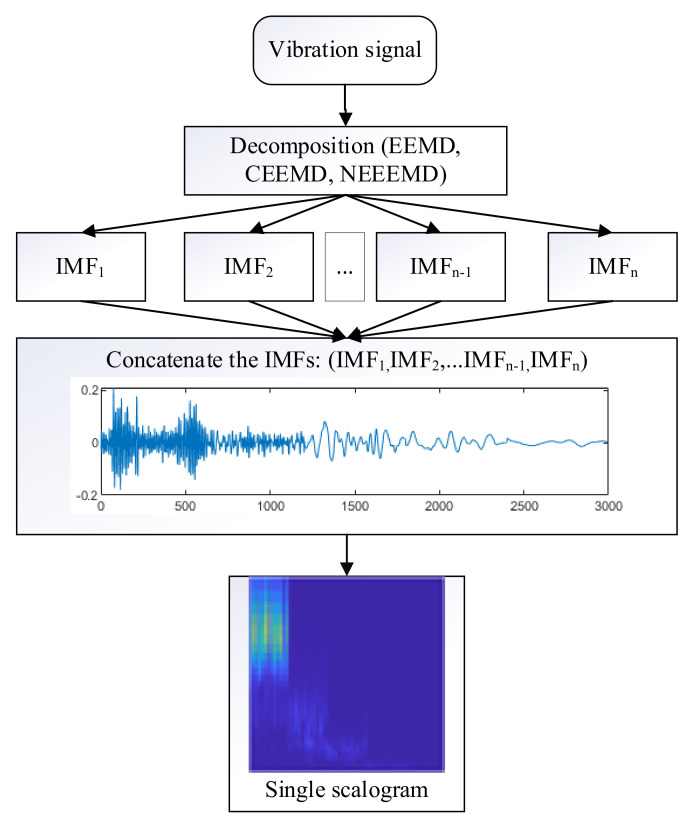
The process of scalogram generation.

Typically, when a signal is decomposed using the adaptive methods, it produces several IMFs. In the previous studies, the authors considered all the individual IMFs as individual samples and applied multichannel CNN for classification [38,39,40]. However, that approach requires *n*-channel CNN for *n*-IMFs, enhancing the preprocessing and classification duration *n* times. For practical application, it is burdensome to do all the computation for the desired outcome. Thus, in this study, all the IMFs have been concatenated and flattened into a single signal. From the single signal, one scalogram image is produced. Thus, the computation time for *n* IMFs was reduced to 1/*n* times. Figure 10 represents the proposed approach for scalogram images input into the classifier model. Here, five IMFs were generated using all the decomposition methods of this study. Concatenating these five IMFs produces a single sample with a length of five times the original sample. Thus, the length of the bearing sample would be 3000, and the blade sample would be 2500. Next, one single scalogram is generated from that sample.

**Figure 11:**
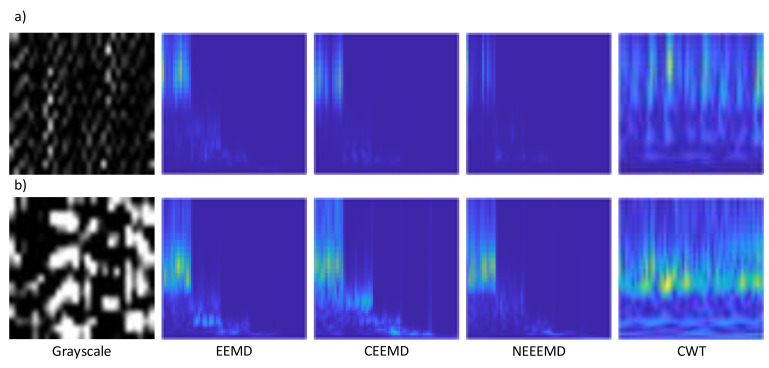
Input samples for deep learning using (**a**) bearing, (**b**) blade dataset.

The colors in the scalogram plot show the relative values of the CWT coefficients. The light areas mean higher values of the CWT coefficients, and therefore, the signal is very similar to the wavelet. On the other hand, dark area means lower values of the CWT coefficients, and it shows that the corresponding time and scale versions of the wavelet are dissimilar to the signal. An RGB (three channels) representation of the time-frequency image is better than a grayscale (one channel) image because more channels contain higher information. Therefore, only RGB scalograms of 64×64 pixels are produced in this study. The scalograms are generated from EEMD, CEEMD, and NEEEMD. Moreover, the scalograms are also obtained from the vibration signals, which are purely CWT scalograms. Figure 11 represents one random sample from each different image type for the bearing and blade dataset. Here, the grayscale samples contain no color values as it has only black and white pixels. Scalograms from the decomposition have five concatenated IMFs, which are clearly visible in their scalogram images. Finally, the CWT scalograms are the pure scalogram samples that are generated from the original sample.

## 7. Deep Learning Classifier

**Figure 12:**
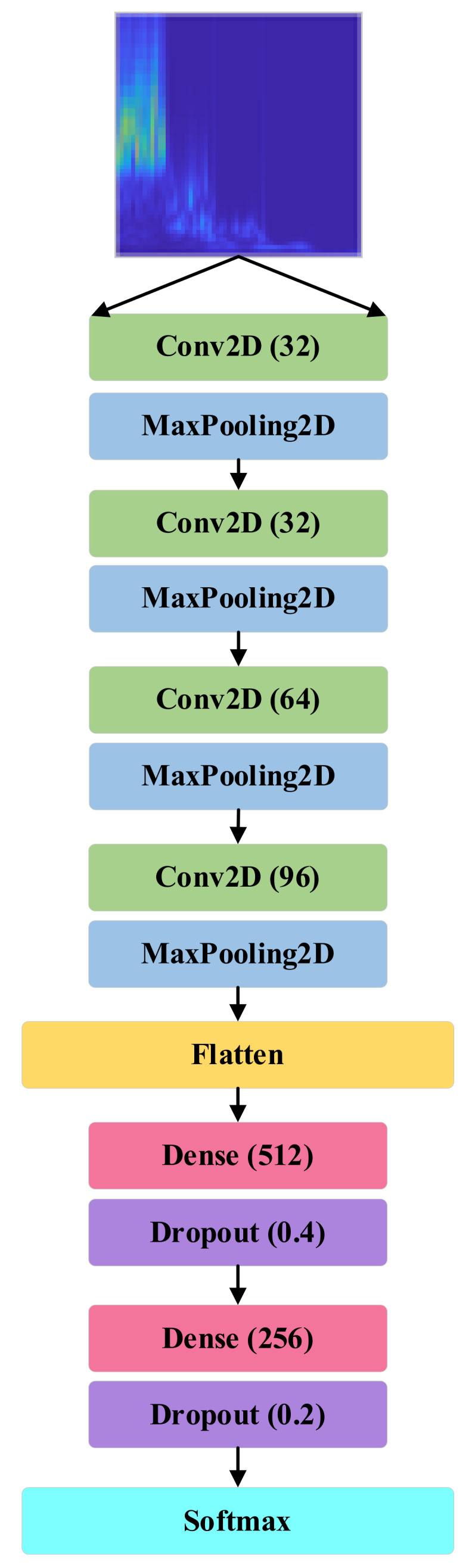
The proposed CNN architecture.

The proposed classifier architecture (Figure 12) consists of only four convolution layers and two dense layers. The input layer of the CNN model consists of three channels that take 64×64 size RGB images. The first convolution layer has 64 output filters with a kernel size of 5×5. The first convolution layer is followed by three other convolution layers where the output filter size is 32, 64, and 96, respectively, and a 3×3 kernel is used in all of them. ‘Same’ padding is used in all these convolution layers. A max-pooling layer of 2×2 pixels is applied after every convolution layer. The output from these three convolution layers is flattened and connected to two dense layers and their respective dropout layers. The first dense layer has 512 neurons with a dropout factor of 0.4 and the second one has 256 neurons with a dropout value of 0.2. ReLU activation function is applied in all of the layers. A softmax activation function is used in the output layer where the layer size is the same as the number of data classes. In the case of binary class, a sigmoid activation function is applied as per convention with only one hidden unit [41]. The bearing model architecture with the number of parameters is presented in Table 4. All models were generated in Python using the Keras library. The total number of parameters and total trainable parameters for this model is 1,006,410.

### Hyperparameters Tuning

Training the CNN model corresponds to a bunch of optimizable parameters. Among the tunable parameters, the most prominent are learning rate, optimizer, loss function, and batch size. An ideal set of these parameter selections for an individual architecture leads to better classification performance. The grid search method was applied in hyperparameter tuning, and the optimum parameters were selected [42].

The deep neural network algorithms use stochastic gradient descent to minimize the error. The learning rate indicates how the model estimates the error each time the weights are updated. Choosing the learning rate is important because a slow learning rate may take a long time to converge, whereas a high learning rate may miss convergence at all. On the other hand, the loss function is used to differentiate the performance of the model between the prediction score and the ground truth. Among many types of loss functions, the cross-entropy loss function is used extraneously and employed in this study.

It is crucial to obtain a low loss using the loss function. To minimize the loss function, implementing an appropriate optimizer is important. An appropriate optimizer sets a bridge between the learning rate and loss function during the gradient descent. The Adam optimizer is an adaptive learning rate optimization algorithm designed especially for DNN. Adam uses momentum in order to accelerate the learning rate when the learning rate becomes slow after a few iterations. The use of Adam can help to prevent being stuck in the local minima of the loss. Adam optimizer is applied to optimize the loss function where the learning rate is set at 0.0001.

The other few important factors of hyperparameters are epoch, early stopping. Epoch refers to how many iterations of forward and backward propagation are conducted before the algorithm is stopped. Another handy trick while training the algorithm is that it is divided into mini-batches instead of taking all the input train samples. This strategy requires less memory usage and trains faster. The early stopping method observes the validation loss or accuracy to monitor if the model is being overfitted. When the model starts to get overfit, the training process is terminated even before the highest number of epochs is reached. In this study, the training data are divided into batches of 16, and the epoch is set at 100. The early stopping criterion is utilized to avoid overfitting the model. As a result, the increase of validation loss is observed for 15 instances, and the best model is saved based on the lowest validation loss. Thus, the overfitting problem is avoided, and the model converges to the minimum loss at an epoch lower than 100 in most cases.

## 8. Data Augmentation Using DCGAN

In this study, DCGAN was utilized because of its ability to use convolution and deconvolution layers in the discriminator and generator to produce high-quality image outputs. The model architecture and the hyperparameters are primarily adopted from the original DCGAN paper [27]. The paper proposed a 64×64×3 RGB image as the input. The DCGAN model has a few deviations from the original GAN. The pooling layers are replaced with convolution layers. Batch normalization is used in the generator and discriminator. LeakyReLu activation function is used in all the layers except for the generator output layer, which is ‘tanh’. The generator model has a 100-dimensional uniform distribution noise vector, Z is given as input.

Some of the hyperparameters are adjusted according to the need for the dataset. All weights were initialized from a zero-cantered normal distribution with a standard deviation of 0.02. In the LeakyReLU, the slope of the leak was set to 0.2 in all models. The Adam optimizer was used with a learning rate of 0.0002 and momentum of 0.5. Binary cross-entropy was used as the loss function. The training samples from each fault type are given into the DCGAN model for the generator to imitate. Since few training samples were available, the batch size while training DCGAN was only 32. It is challenging to generate fake samples using only a few training data. Thus, 10,000 epochs were used to train the model, and the desired number of fake samples were generated once the highest number of epochs was reached.

## 9. Outputs from the Classifiers

This section compares outputs from NEEEMD scalograms classification using CNN with related scalograms and grayscale images. The train, validation, and test accuracies are obtained for comparison. Moreover, the individual class performance from the test results is used to obtain the sensitivity values for comparison. The sensitivity values are considered because this study emphasizes mainly the correct identification of fault types.

Table 5 represents the output accuracy from the bearing fault classifiers, and Table 6 represents its class sensitivity. The order of validation and test accuracies for the CNN classifiers are grayscale, CWT, EEMD, CEEMD, and NEEEMD scalograms. Here, the lowest validation and test accuracies are 95.60% and 94.40%, obtained by the grayscale images. The highest validation and test accuracy obtained by NEEEMD scalograms are 98.20% and 98%. The sensitivity values show that NEEEMD scalograms obtained the highest sensitivity for each individual class. In other methods, some classes obtained the highest sensitivity but not for all classes. Therefore, all the classes had the most correctly identified fault occurrence using the NEEEMD scalogram samples for the bearing classifier.

The output accuracy and sensitivity for the blade fault diagnosis classifier are presented in Table 7 and Table 8. The order of the validation and test accuracy is similar to the order of bearing fault classifier output. All the output accuracies of the scalograms had small differences among train, validation, and test accuracy, indicating a well-trained classifier. However, the validation and test accuracies had comparatively lower accuracy than the training accuracy in grayscale samples. Therefore, the classifier trained on grayscale samples was more overfit than the others. EEMD obtained slightly higher accuracy than CWT scalograms, and the same goes for CEEMD when compared with EEMD. NEEEMD scalograms obtained the highest validation and test accuracies, which were 97.84% and 96.31%. All the output accuracies are reflected in the sensitivity of classes. In grayscale samples, a large proportion of class 0 was misclassified. On the other hand, the NEEEMD scalogram classifier had the highest individual class sensitivity.

Table 9 and Table 10 list the output accuracy and sensitivity from blade fault localize classifiers. The NEEEMD scalograms obtained the highest validation and test accuracy for CNN as well. However, the grayscale and CWT samples obtained a highly overfit model. The validation and test accuracies deviate much from the training accuracy. The grayscale samples had a train, validation, and test accuracy of 98.38%, 71.75%, and 59.81%. For the CWT scalograms, the train, validation, and test accuracies are 95.98%, 75.17%, and 68.44%. These outputs indicate the poor performance of grayscale and CWT scalograms for a higher-class classification. On the other hand, the scalogram samples from EEMD, CEEMD, and NEEEMD obtained much less overfit classifier as the validation and test accuracies were very close to the training accuracy. NEEEMD obtained the highest validation and test accuracies, 93.27%, and 92.25%, respectively. In terms of sensitivity, a few classes from grayscale and CWT samples are below 0.50, meaning more samples were misclassified and correctly classified. On the other hand, NEEEMD had the highest sensitivity for all individual classes. Therefore, the NEEEMD scalogram can still obtain very high accuracy and sensitivity for a higher-class classification.

This section shows that the combination of NEEEMD scalograms and the CNN model performed the best. The grayscale samples and CNN. In most of the scalograms and CNN models, the output performance was very close to each other. Thus, more analysis is needed for additional justification of the best model for different situations. Therefore, all of the scalogram classification models will be undertaken for further evaluation using model robustness for noisy test samples.

### 9.1. Robustness Evaluation

When predicting new data, the data can be quite deviated from the original signal, or they may contain high noise in the real application. Therefore, it is essential to ensure that the model can still hold good robustness when classifying new data. Gaussian white noise is added with the test samples and it is predicted using the trained model to verify the robustness of all the models. The white noise is added directly to the scalogram images (as salt-pepper noise) and incremented at a step of 0.05 standard deviation (SD). The outputs from the scalogram samples of NEEEMD, CEEMD, EEMD, and CWT are compared. The grayscale vibration image classification using CNN and the machine learning classifiers are not considered as they already performed with considerably low accuracy in the earlier stage. The outputs from all the datasets and classifiers are obtained in the following figures.

**Figure 13:**
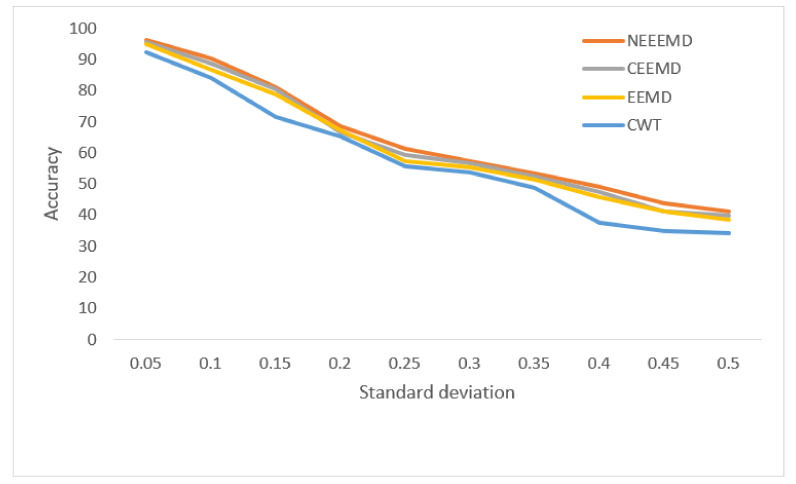
Performance of bearing fault classifier on noisy data.

**Figure 14:**
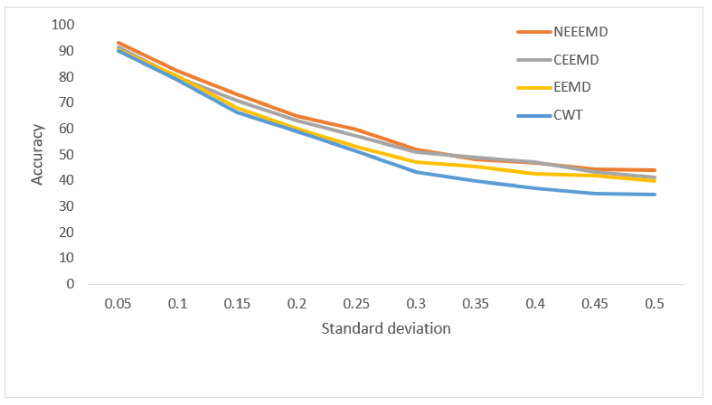
Performance of blade fault diagnosis classifier on noisy data.

**Figure 15:**
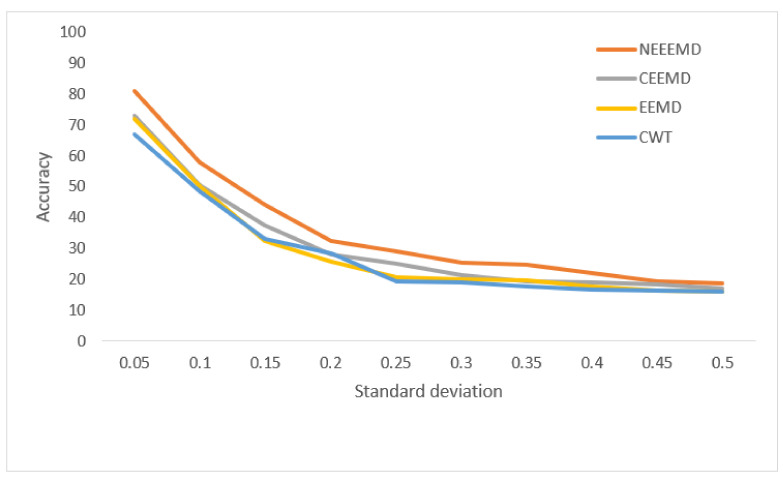
Performance of blade fault localization classifier on noisy data.

In Figure 13, the bearing test dataset with different noise levels shows accuracies in the descending order of NEEEMD, CEEMD, EEMD, and CWT. The CWT samples model’s robustness keeps falling most for a higher order of noise. The NEEEMD, CEEMD, and EEMD models’ robustness are close, but the NEEEMD model could maintain the highest robustness all the way. In Figure 14, the models’ robustness for the fault diagnosis classifier is obtained. The robustness of CWT was the lowest for all the SD, followed by EEMD. The robustness of NEEEMD and CEEMD are very close. The robustness of NEEEMD is higher than CEEMD for all the SDs except for only 0.35 SD, where it lags by a tiny portion. Apart from that, the NEEEMD samples for the fault diagnosis model performed with the highest robustness. In Figure 15, the differences in robustness for the fault localize classifiers were much higher in the earlier stages of noise SDs. The robustness of NEEEMD is much higher than all the other comparable methods. However, at the highest level of noise SD, these performance differences shrink. Nevertheless, for different noise SD levels, the accuracy curve of CEEMD, EEMD, CWT overlap at some point. Meaning, these three models’ order of robustness varies at different noise SDs. On the other hand, the accuracies of the NEEEMD model are comparatively higher than the other scalogram samples. These outputs indicate that the proposed NEEEMD scalograms with CNN classifier can provide the highest robustness than the other methods considered in this study.

### 9.2. Performance with Augmented Samples

**Figure 16:**
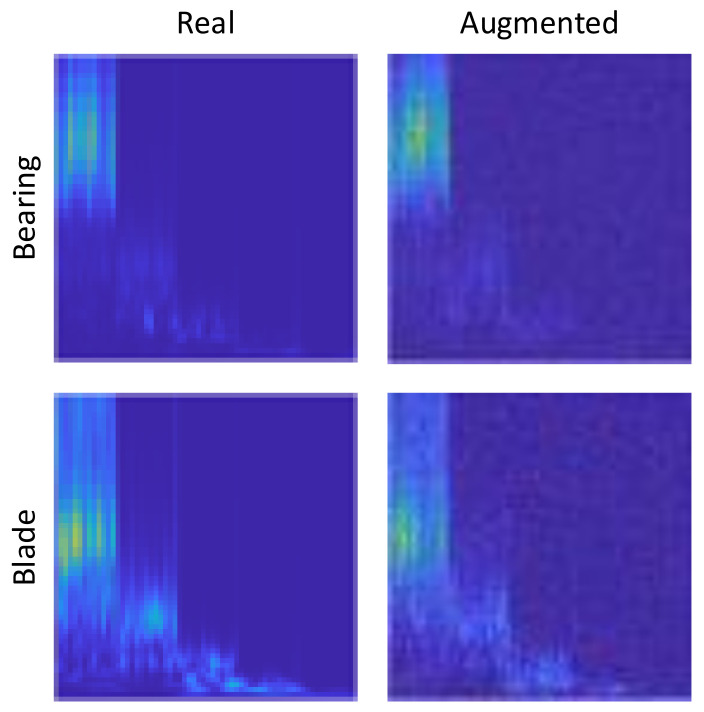
Augmented image samples from DCGAN.

Now that it is established that the proposed NEEEMD scalograms + CNN models performed better than all the other methods, additional improvement in performance is conducted. The goal is to increase the number of training samples with the augmented data to investigate how the classifier performance changes. The fake samples from DCGAN output show that the fake samples could significantly mimic the original samples of the respective classes. However, DCGAN takes in a complete white noise to perform augmentation, and the presence of some noise can still be seen in the output samples. How to further improve this output and reduce noise is another research problem. For the classifier models, it can be predicted that adding the augmented samples in proportion with the original training samples should somewhat improve the accuracy. Nevertheless, populating the classifier with too many fake samples might have a reverse effect because the classifier would learn most from the fake data. DCGAN is applied to the samples from every single class of bearing and blade data. The desired amount of augmented samples are generated during the process. One random output of bearing and blade data using DCGAN are presented in Figure 16 for visualization. The augmented samples have some degree of white noise presented in them as it is completely generated from random white noise. However, the augmented scalograms still successfully very much mimic the real scalograms.

In [43], the authors attempted to find the relation between classifier accuracy and the train size. They considered three different CNN classifier models from different literature and gradually increased the training accuracy. It is observed that as the training size is increased, the accuracy also improves. However, after a turning point, the accuracy and model robustness are decreased. Moreover, it is established that for DNN, there is no definite right number of samples as the right number depends on the types of data and model architecture. It shows how the accuracy is the highest at the turning point, and the number of samples is the right number of samples for the model.

**Figure 17:**
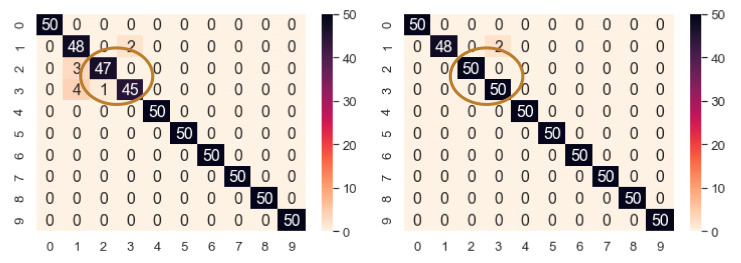
Confusion matrix of NEEEMD and NEEEMD + DCGAN at the turning point for bearing fault classification.

The bearing dataset has only 100 training samples. To observe how the model reacts to the increased training samples, the augmented samples are increased at a step of 50 for each class. Thus, for each increment, 500 new fake samples are added. The accuracy obtained using fake samples from DCGAN is listed in Table 11. As the number of training samples is increased, the validation and test accuracy also increase. The highest validation and test accuracy are obtained at fake samples of 150 per class, i.e., a total of 2500 training samples. This is the turning point of our classifier model for the increased samples, and beyond this point, the accuracy keeps falling gradually. Therefore, the validation and test accuracy are enhanced from 98.2% to 99.6% and 98% to 99.6%, respectively, whereas it falls to 97.4% and 97.2%, respectively. Figure 17 shows the performance improvement of NEEEMD + DCGAN from NEEEMD only. In only NEEEMD scalograms output, all the inner race fault classes had several misclassified samples. On the other hand, in NEEEMD + DCGAN output, only class 1 and class 2 contain misclassified samples. The higher fault severity of inner race, i.e., class 2 and class 3 had all correctly classified samples. Therefore, apart from class 1, all the other classes of NEEEMD + DCGAN were correctly classified. Thus, it can be concluded that, DCGAN improved the classifier robustness and produced more correctly classified samples.

**Figure 18:**
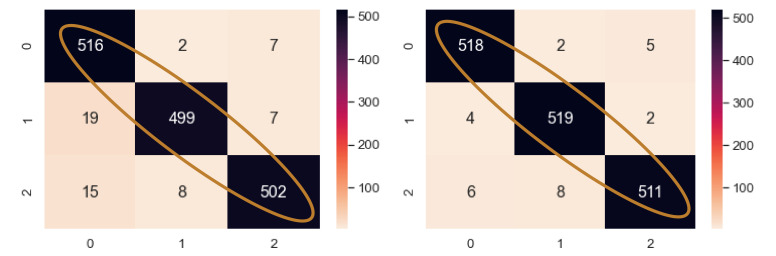
Confusion matrix of NEEEMD and NEEEMD + DCGAN at the turning point for blade fault diagnosis.

The fault diagnosis model has 3 fault classes, each containing 1750 samples. The fake samples are added in a batch of 100 for each class. It means that for 3 classes, 300 samples are added with the training data in each step. At around 300 fake samples for each class, i.e., 900 more added samples and 5250 original samples (6150 total samples), the classifier reaches its turning point and achieves the highest accuracy, as shown in Table 12. The validation and test accuracy rose to 98.60% and 98.29% from 97.84% and 96.31%, respectively, and again fell to 96.76% and 95.24%. The confusion matrix for the 3-class classifier is shown in Figure 18. Here, all the classes had more correctly classified samples than the previous model with no augmentation. Class 0, 1, and 2 had 2, 20, and 9 more correctly classified samples, respectively, than the previous best model. As a result, the sensitivity for each class of the NEEEMD + DCGAN improved.

**Figure 19:**
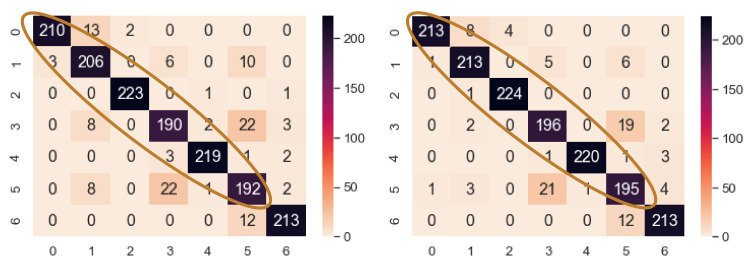
Confusion matrix of NEEEMD and NEEEMD + DCGAN at the turning point for blade fault localization.

The fault localize model has 7 fault classes, each containing 750 samples. The fake samples are added in a batch of 100 for each class. This means that for 7 classes, 700 samples are added with the training data in each step. From Table 13, around 400 fake samples for each class, i.e., 2800 more added samples and 5250 original samples (total 8050 training samples), the classifier reaches its turning point and achieves the highest accuracy. The validation and test accuracy raise to highest 94.67% and 93.59% from 93.27% and 92.25%, respectively, and again falls to 91.68% and 89.84%. The confusion matrix is shown in Figure 19. Here, only class 6 had the same number of correctly classified samples in both cases. All the other classes in NEEEMD + DCGAN contain higher classified samples than NEEEMD only. The number of increased correctly classified samples are 3, 7, 1, 6, 1, and 3, in the order of the classes, respectively.

The improved performance using NEEEMD and DCGAN samples at the turning point is compared with the NEEEMD scalograms + CNN output and presented in Table 14. The train, validation, test accuracies, and sensitivity values are considered to compare the bearing fault classification and three blade fault classifications. All four models show impressive improvements in validation and test accuracies. The sensitivity values for the classes are also obtained. All of the classes from NEEEMD + DCGAN show higher sensitivity values than the NEEEMD samples. Therefore, the fake samples generated from the DCGAN help to increase the CNN performance.

### 9.3. Improvement in Robustness

For all cases, the model accuracy increases up to a point with the increment of training samples. It can be concluded that the right amount of training samples will make the classifier more accurate. However, in order to develop a more robust model, some researchers tried to add some degree of white noise to the training data during the training phase. This technique also helps to reduce the overfitting problem. It first showed the effect of added white noise during backpropagation for a more generalized model [44]. It is found that the input noise is effective for a more generalized classification and regression model. Kosko et al. showed that noise could generalize the classifier as well as speed up backpropagation learning [45]. The augmented samples using DCGAN has some presence of noise in all of them. Since these noisy augmented samples are incorporated with the other training samples, a more robust classifier is expected. Gaussian white noise is added with the test samples and predicted using the trained model to verify our hypothesis. The outputs from the scalogram samples of NEEEMD + DCGAN are compared with NEEEMD to observe the performance improvement. The outputs from all the datasets and classifiers are obtained in the following figures.

**Figure 20:**
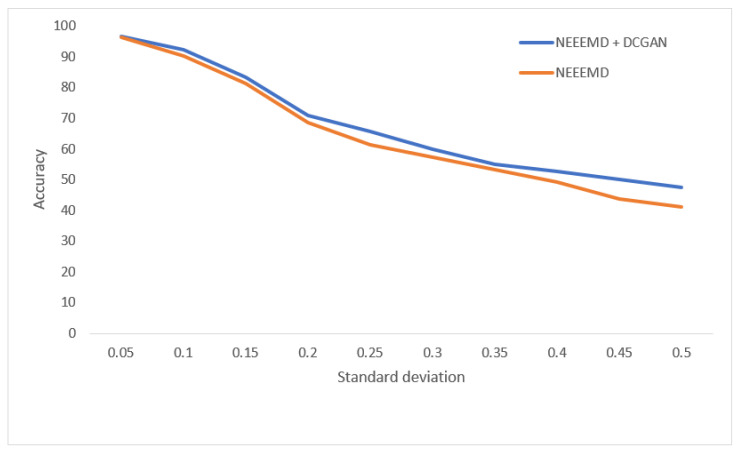
Performance of bearing fault classifier on noisy data.

**Figure 21:**
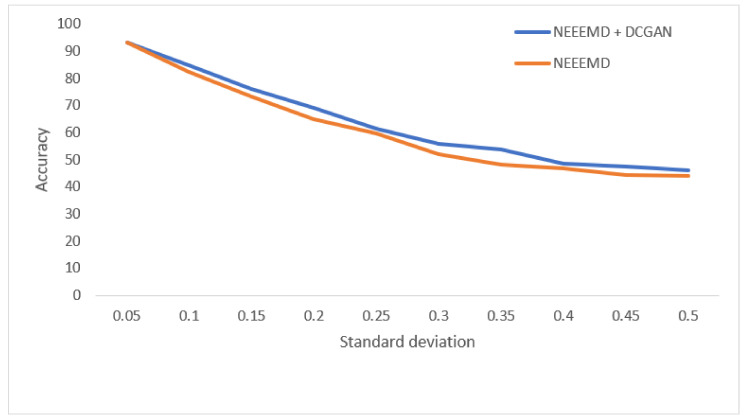
Performance of blade fault diagnosis classifier on noisy data.

**Figure 22:**
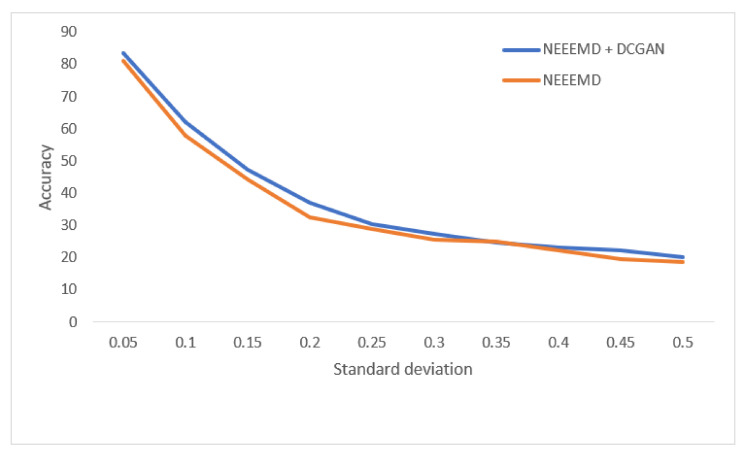
Performance of blade fault localization classifier on noisy data.

In Figure 20, for the bearing fault classifier, as the noise SD levels rise, the NEEEMD + DCGAN model performed with significantly higher robustness than the NEEEMD scalograms model. In Figure 21, for the blade fault diagnosis model, the NEEEMD + DCGAN model maintains relatively constant higher robustness than the NEEEMD model. In Figure 22, the fault localization model for NEEEMD + DCGAN has higher robustness at all the noise SDs except 0.35 SD. At 0.35 SD, the robustness is the same for both NEEEMD + DCGAN and NEEEMD samples only. Apart from this only exception, the NEEEMD + DCGAN model performed with considerably higher robustness in all the classifier models. This proves that the augmented samples from DCGAN not only improved our classification accuracy, but also enhanced the models’ robustness.

## 10. Conclusions

In this work, the effectiveness of NEEEMD scalograms is verified by classifying using CNN and compared with scalograms from CWT, EEMD, CEEMD, and grayscale images. The scalogram samples obtained much higher accuracy than grayscale image samples. The NEEEMD scalograms obtained the highest accuracy in all the classifier models. On the other hand, the grayscale images and CWT scalograms obtained an overfit model for a higher-class classification. Nevertheless, the outputs of the scalograms samples had a small difference in terms of accuracy. Therefore, additional validation is conducted by considering model robustness for noisy test samples. The NEEEMD scalograms and CNN model obtained the highest robustness in all the methods. Next, augmented samples from DCGAN are used with the original samples to train the NEEEMD scalograms and CNN classifier again. The number of augmented samples is increased within a range to observe how the classifier performance varies. Thus, the optimal number of training samples is obtained where the classifier obtained improved validation and accuracy. The model robustness at this point is compared with the previous robustness from NEEEMD scalograms and CNN. It is found that the augmented samples from DCGAN improve classifier accuracy and increase model robustness.

## Figures and Tables

**Table 1:** Different bearing fault conditions considered in this study.

Fault	Normal	Inner Race			Outer Race			Ball		
Class	0	1	2	3	4	5	6	7	8	9
Severity	N/A	0.007″	0.014″	0.021″	0.007″	0.014″	0.021″	0.007″	0.014″	0.021″

**Table 2:** Datasets for the blade fault diagnosis classifier.

Type	Rub	Loss	Twist
Class	0	1	2
Data	BR-R1	BL-R1	BT-R1
	BR-R2	BL-R2	BT-R2
	BR-R3	BL-R3	BT-R3
	BR-R12	BL-R12	BT-R12
	BR-R13	BL-R13	BT-R13
	BR-R23	BL-R23	BT-R23
	BR-R123	BL-R123	BT-R123

**Table 3:** Datasets for the blade fault localization classifier.

Type	Row 1	Row 2	Row 3	Row 1,2	Row 1,3	Row 2,3	Row 1,2,3
Class	0	1	2	3	4	5	6
Data	BR-R1	BR-R2	BR-R3	BR-R12	BR-R13	BR-R23	BR-R123
	BL-R1	BL-R2	BL-R3	BL-R12	BL-R13	BL-R23	BL-R123
	BT-R1	BT-R2	BT-R3	BT-R12	BT-R13	BT-R23	BT-R123

**Table 4:** Parameters of the proposed CNN model.

Model: “Sequential”		
Layer (type)	Output Shape	Parameter #
conv2d (Conv2D)	(None, 64, 64, 32)	2432
max_pooling2d (MaxPooling2D)	(None, 32, 32, 32)	0
conv2d_1 (Conv2D)	(None, 32, 32, 32)	9248
max_pooling2d_1 (MaxPooling2D)	(None, 16, 16, 32)	0
conv2d_2 (Conv2D)	(None, 16, 16, 64)	18,496
max_pooling2d_3 (MaxPooling2D)	(None, 8, 8, 64)	0
conv2d_3 (Conv2D)	(None, 8, 8, 96)	55,392
max_pooling2d_3 (MaxPooling2D)	(None, 4, 4, 96)	0
flatten (Flatten)	(None, 1536)	0
dense (Dense)	(None, 512)	786,944
dropout (Dropout)	(None, 512)	0
dense_1 (Dense)	(None, 256)	131,328
dropout_1 (Dropout)	(None, 256)	0
dense_2 (Dense)	(None, 10)	2570
Total parameters: 1,006,410 Trainable parameters: 1,006,410 Non-trainable parameters: 0		

**Table 5:** All undertaken classifiers’ accuracy for bearing fault classification.

Method	Accuracy		
	Train (%)	Validation (%)	Test (%)
Grayscale image + CNN	99.10	95.60	94.40
CWT scalogram + CNN	98.20	97.60	97
EEMD scalogram + CNN	98.60	97.60	97.20
CEEMD scalogram + CNN	98.40	98	97.60
NEEEMD scalogram + CNN	98.40	98.20	98

**Table 6:** All undertaken classifiers’ sensitivity values for bearing fault classification.

Method	Sensitivity									
Class	0	1	2	3	4	5	6	7	8	9
Grayscale image + CNN	1	0.84	0.88	0.9	1	0.86	1	1	0.96	1
CWT scalogram + CNN	1	0.9	0.9	0.9	1	1	1	1	1	1
EEMD scalogram + CNN	1	0.92	0.9	0.9	1	1	1	1	1	1
CEEMD scalogram + CNN	1	0.92	0.92	0.9	1	1	1	1	1	1
NEEEMD scalogram + CNN	1	0.96	0.94	0.9	1	1	1	1	1	1

**Table 7:** All undertaken classifiers’ accuracy for blade fault diagnosis classification.

Method	Accuracy		
	Train (%)	Validation (%)	Test (%)
Grayscale image + CNN	96.32	88.63	83.49
CWT scalogram + CNN	99.30	96.83	94.35
EEMD scalogram + CNN	99.64	97.33	94.66
CEEMD scalogram + CNN	99.67	97.59	95.37
NEEEMD scalogram + CNN	99.42	97.84	96.31

**Table 8:** All undertaken classifiers’ sensitivity values for fault diagnosis classification.

Method	Sensitivity		
Class	0	1	2
Grayscale image + CNN	0.739	0.922	0.844
CWT scalogram + CNN	0.95	0.933	0.947
EEMD scalogram + CNN	0.952	0.933	0.954
CEEMD scalogram + CNN	0.968	0.939	0.954
NEEEMD scalogram + CNN	0.983	0.95	0.956

**Table 9:** All undertaken classifiers’ accuracy for blade fault localization classification.

Method	Accuracy		
	Train (%)	Validation (%)	Test (%)
Grayscale image + CNN	98.38	71.75	59.81
CWT scalogram + CNN	95.98	75.17	68.44
EEMD scalogram + CNN	98.15	91.87	91.05
CEEMD scalogram + CNN	98.35	92.13	91.68
NEEEMD scalogram + CNN	97.59	93.27	92.25

**Table 10:** All undertaken classifiers’ sensitivity values for blade fault localization classification.

Method	Sensitivity						
Class	0	1	2	3	4	5	6
Grayscale image + CNN	0.858	0.422	0.871	0.827	0.524	0.347	0.338
CWT scalogram + CNN	0.693	0.56	0.56	0.729	0.827	0.613	0.809
EEMD scalogram + CNN	0.924	0.907	0.96	0.827	0.929	0.849	0.933
CEEMD scalogram + CNN	0.924	0.911	0.991	0.836	0.964	0.853	0.938
NEEEMD scalogram + CNN	0.933	0.916	0.991	0.844	0.973	0.853	0.947

**Table 11:** Performance of bearing fault classifier for different numbers of augmented training samples (optimal number highlighted in bold).

Fake Data/Class	Accuracy		
	Train (%)	Validation (%)	Test (%)
No fake	98.40	98.20	98
50	99.0	98.6	98.2
100	99.6	98.8	98.8
**150**	**99.69**	**99.60**	**99.60**
200	98.33	98.2	98
250	98.93	98	98
300	98.65	97.8	97.8
350	99.24	97.8	97.6
400	99.62	97.8	97.4
450	98.62	97.4	97.4
500	99.71	97.4	97.2

**Table 12:** Performance of blade fault diagnosis classifier for different numbers of augmented training samples (optimal number highlighted in bold).

Fake Data/Class	Accuracy		
	Train (%)	Validation (%)	Test (%)
No fake	99.42	97.84	96.31
100	99.33	98.03	96.76
200	99.63	98.03	97.21
**300**	**99.63**	**98.60**	**98.29**
400	99.96	98.16	98.03
500	99.66	97.90	96.89
600	99.46	97.59	96.70
700	99.91	97.46	96.19
800	99.23	97.08	96.00
900	99.69	96.83	95.24
1000	99.04	96.76	95.24

**Table 13:** Performance of blade fault localization classifier for different numbers of augmented training samples (optimal number highlighted in bold).

Fake Data/Class	Accuracy		
	Train (%)	Validation (%)	Test (%)
No fake	97.59	93.27	92.25
100	96.62	93.46	92.57
200	97.85	93.78	93.21
300	98.61	93.97	93.27
**400**	**98.06**	**94.67**	**93.59**
500	98.05	93.40	92.19
600	97.85	93.33	91.68
700	98.35	92.95	91.68
800	96.88	92.95	90.79
900	97.13	92.32	90.48
1000	98.56	91.68	89.84

**Table 14:** Improvement in classifiers’ performance using the augmented training samples from DCGAN.

Model	CNN Input											
Bearing fault	NEEEMD (Samples: 1000)	Accuracy	Train: 98.40%, validation: 98.20%, test: 98%									
		Class	0	1	2	3	4	5	6	7	8	9
		Sensitivity	1	0.96	0.94	0.9	1	1	1	1	1	1
	NEEEMD + DCGAN (Samples: 2500)	Accuracy	Train: 99.69%, validation: 99.6%, test: 99.6%									
		Sensitivity	0	1	2	3	4	5	6	7	8	9
		Class	1	0.96	1	1	1	1	1	1	1	1
Blade fault diagnosis	NEEEMD (Samples: 5250)	Accuracy	Train: 99.42%, Val: 97.84%, Test: 96.31%									
		Class	0				1			2		
		Sensitivity	0.983				0.95			0.956		
	NEEEMD + DCGAN (Samples: 6150)	Accuracy	Train: 99.63%, Val: 98.60%, Test: 98.29%									
		Class	0				1			2		
		Sensitivity	0.987				0.989			0.973		
Blade fault localization	NEEEMD (Samples: 5250)	Accuracy	Train: 97.59%, Val: 93.27%, Test: 92.25%									
		Class	0	1		2	3		4	5		6
		Sensitivity	0.933	0.916		0.991	0.844		0.973	0.853		0.947
	NEEEMD + DCGAN (Samples: 8050)	Accuracy	Train: 98.06%, validation: 94.67%, test: 93.59%									
		Class	0	1		2	3		4	5		6
		Sensitivity	0.947	0.947		0.996	0.895		0.978	0.867		0.947

## Supplementary Materials

All of the coding files used to obtain the results of this study can be found at https://github.com/atik666/Data-Final.

## Appendix A

### Appendix A.1. EMD

The time-frequency domain analysis EMD simply decomposes a vibration signal into several IMFs. The IMF function needs to fulfill two criteria:

1. The number of extrema and zero crossings in the whole vibration series must be the same or differ at most by one.
2. The mean of upper and lower envelopes must be zero at any given point.

When the above two criteria are satisfied, an IMF is reported by 𝑐_1_. The first residue $r_{1}\;$ is obtained from the difference between the signal and the first IMF and is used to decompose the next IMF. The decomposition process has to be repeated to find the 𝑛th IMF until a monotonic residue is reported. The method of decomposition is repeated n times until the residue becomes monotonic to obtain the nth IMF. The original signal can be reassembled by summing the 𝑛 IMFs and the 𝑛th residue presented as follows:

$$X\left(t\right)=\overset{n}{\underset{i=1}{\sum }}c_{i}+r_{n}$$

where $X\left(t\right)$ is the original signal, 𝑐_i_ is the 𝑖th IMF, and 𝑟_n_ is the 𝑛th residue.

### Appendix A.2. EEMD

EEMD is an improvement of EMD, which was suggested to solve the mode-fusing problem of EMD. The white noise of a finite amplitude is added with the ensembles of EMD to generate the IMFs. The added noise dissolves in the whole time-frequency plane uniformly, thus solving the mode-mixing problem. For an original signal, $X\left(t\right),\;$ the EEMD algorithm follows the following steps:

1. Add a bunch of white noise to the original signal with a mean of 0 and the standard deviation of 1 to obtain a series of ensembles.
  $$X_{i}\left(t\right)=X\left(t\right)+w_{i}\left(t\right)$$
  where $w_{i}\left(t\right)$ is the white noise signal of the same length as $x\left(t\right)$ and i=1,2, …, M, M is the number of ensembles.
2. Decompose the ensembles using EMD to obtain the IMFs.
  $$X_{i}\left(t\right)=\overset{N}{\underset{j=1}{\sum }}c_{ij}\left(t\right)+r_{i}\left(t\right)$$
  where j=1,2,…,N, N is the number of IMFs and $c_{ij}\left(t\right)$ is the IMFs ($c_{i1},\;c_{i2},\ldots ,c_{iN}$). $r_{i}\left(t\right)$ denotes the residue of the ith trail.
3. In the end, determine the ensemble means of the consequent IMFs.
  $$c_{j}\left(t\right)=\frac{1}{M}\overset{M}{\underset{i=1}{\sum }}c_{ij}\left(t\right)$$

### Appendix A.3. CEEMD

CEEMD was proposed to overcome the drawbacks and increase the performance of EEMD. In CEEMD, white noise is added in pairs (both positive and negative) to reduce the presence of noise. The paired white noises can decrease the white noise in the final residue successfully. The CEEMD algorithm for a signal $X\left(t\right)$ has the following steps:

1. Add a pair of white noise to $x\left(t\right)$ the same way as EEMD.
  $$X_{1}\left(t\right)=X\left(t\right)+w_{i}\left(t\right)\;X_{2}\left(t\right)=X\left(t\right)-w_{i}\left(t\right)$$
2. Decompose $X_{1}\left(t\right)$ and $X_{2}\left(t\right)$ using EMD to obtain the IMFs.
3. Obtain two ensemble IMFs sets by repeating the steps M times.
  $$IMF_{1}=\frac{1}{M}\overset{M}{\underset{i=1}{\sum }}IMF_{1i}\;IMF_{2}=\frac{1}{M}\overset{M}{\underset{i=1}{\sum }}IMF_{2i}$$
4. The final IMFs are obtained from the mean of positive and negative ensembles.
  $$IMF=\left(IMF_{1}+IMF_{2}\right)/2$$
5. The final result is obtained as,
  $$X\left(t\right)=\overset{M}{\underset{i=1}{\sum }}IMF_{i}\left(t\right)+r_{M}\left(t\right)$$
